# Widespread Functional and Structural Dysconnectivity Patterns in Treatment-Resistant Schizophrenia

**DOI:** 10.1192/j.eurpsy.2025.1671

**Published:** 2025-08-26

**Authors:** L. Z. Ueta, C. E. G. Salmon, R. D. C. Correia, J. M. Ushirohira, A. C. Santos, J. E. C. Hallak

**Affiliations:** 1 University of Sao Paulo, Ribeirao Preto, Brazil

## Abstract

**Introduction:**

Treatment-resistant schizophrenia patients have the worst clinical outcomes and poor quality of life, despite the administration of clozapine. Currently, the definition of resistance is based on treatment response, but evidence differentiating resistance remains unclear. The dysconnectivity theory suggests that disrupted brain interactions contribute to schizophrenia pathology. Advances in functional and structural neuroimaging allow exploration of connectivity patterns that may characterize treatment-resistant schizophrenia.

**Objectives:**

This study aimed to describe functional and structural connectivity patterns in schizophrenia patients on clozapine.

**Methods:**

Patients taking clozapine (CLZ) or other antipsychotic (nCLZ) were recruited from the Hospital of the Medical School of Ribeirão Preto. Healthy controls (HC) were recruited from the Hospital database. Seventy-seven participants were selected: CLZ = 35, nCLZ = 27, and HC = 27. Functional connectivity (FC) was assessed via resting-state functional magnetic resonance imaging (rs-fMRI) and ROI-to-ROI analysis using the CONN toolbox. Structural connectivity was analyzed with diffusion tensor imaging and tractography using TRACULA toolbox. ANOVA and Tukey test were used for three-group comparisons, and the t-tests for two-group comparisons. Functional analysis was performed with a significance threshold of p < 0.05 and false discovery rate (FDR) set to p < 0.01.

**Results:**

Reduced frontotemporal FC and increased FC in the frontal-occipital were common in CLZ and nCLZ compared to HC. An increased FC between sensorimotor and the cerebellum was notable in CLZ compared to HC. Structural findings included increased axial diffusivity (AD) and mean diffusivity (MD) in 8 of 10 tracts in CLZ compared to HC. The corticospinal tract and inferior longitudinal fasciculus exhibited increased AD and MD in CLZ compared to nCLZ. The cingulum angular bundle showed significantly altered diffusion measures in nCLZ: increased AD, MD, and radial diffusivity (RD), and reduced fractional anisotropy (FA).

**Conclusions:**

This study described a widespread functional and structural dysconnectivity in CLZ, characterized by reduced frontotemporal FC and increased frontal-occipital and sensorimotor–cerebellum FC. Altered AD and MD measures in the corticospinal tract and inferior longitudinal fasciculus were observed in the CLZ. Alterations in AD, MD, RD, and FA measures in the cingulum angular bundle, particularly noted in nCLZ, may be associated with antipsychotic administration. Further studies are necessary to establish whether these dysconnectivity patterns consistently characterize these patient groups.

**Disclosure of Interest:**

None Declared

